# Differential mechanisms of action understanding in left and right handed subjects: the role of perspective and handedness

**DOI:** 10.3389/fpsyg.2013.00957

**Published:** 2013-12-19

**Authors:** Rachel L. Kelly, Lewis A. Wheaton

**Affiliations:** Cognitive Motor Control Laboratory, School of Applied Physiology, Georgia Institute of TechnologyAtlanta, GA, USA

**Keywords:** perspective, handedness, action understanding, limb-matched, mirror-matched, tool use

## Abstract

The ability to comprehend outcomes of skilled action is important for understanding the world around us. Prior studies have evaluated the perspective an action is performed in, but few have evaluated how handedness of the actor and the observer interact with action perspective. Understanding handedness affords the opportunity to identify the role of mirroring and matched limb action encoding, which may display unique strategies of action understanding. Right and left-handed subjects were presented with images of tools from egocentric or allocentric perspectives performing movements by either a left or right hand. Subjects had to judge the outcome of the task, and accuracy and latency were evaluated. Our hypothesis was that both left and right-handed subjects would predict action best from an egocentric perspective. In allocentric perspectives, identification of action outcomes would occur best in the mirror-matched dominant limb for all subjects. Results showed there was a significant effect on accuracy and latency with respect to perspective for both right and left-handed subjects. The highest accuracies and fastest latencies were found in the egocentric perspective. Handedness of subject also showed an effect on accuracy, where right-handed subjects were significantly more accurate in the task than left-handed subjects. An interaction effect revealed that left-handed subjects were less accurate at judging images from an allocentric viewpoint compared to all other conditions. These findings suggest that action outcomes are best facilitated in an internal perspective, regardless of the hand being used. The decreased accuracy for left-handed subjects on allocentric images could be due to asymmetrical lateralization of encoding action and motoric dominance, which may interfere with translating allocentric limb action outcomes. Further neurophysiological studies will help us understand the specific processes of how left and right-handed subjects may encode actions.

## INTRODUCTION

Understanding skilled action is a basic aspect of our daily living. Skilled action in humans frequently involves the use of tools in order to complete action goals. In order to understand skilled tool-use actions, we must understand at least two elements: how to identify the tool needed for a specific task (contextual knowledge) and understand how the tool is used to complete the action goal (physical knowledge; Mizelle and Wheaton, 2010). Our knowledge of a tool comes from the fact that we learn tool and action associations in our cognitive-motor system and from this knowledge we are able to use it to understand not only how to accomplish skilled actions ourselves, but also how to predict the ultimate goal of actions executed by others.

Previous research suggests how action understanding occurs through observation (Fadiga et al., 1995; Iacoboni et al., 1999; Bekkering et al., 2000; Rizzolatti et al., 2004). Action understanding likely requires an imitative capability that allows a persons’ motor system to precisely organize body motion in order to achieve an observed movement. The ideomotor theory describes that action and the perception of action are related by common neural systems (Massen and Prinz, 2009). Thus perceiving another’s actions or action outcomes elicits the same action in the observer’s motor system. It has been proposed that when viewing a tool or object, not only are the physical elements of the scene being processed, but also an additional higher level of processing occurs which can prompt “functional affordance” representations (Mizelle et al., 2013). In this work, functional affordances are the possible object-based tool actions that best “afford” a desired action goal. When subjects looked at static correct tool and object images, sensorimotor activation was observed which indicated that action was being understood and the motor system was being driven. Type of tool or object also affects the ability to understand the ultimate action goal. New tools might not be able to simulate a motor plan as would a known tool; however, our previous work (Mizelle et al., 2011) has shown that after directly training with a novel tool one time, it activates the same neural tool network that known tools activate.

Seeing an action and being able to recognize the possible outcomes are vital for not only the potential of motor simulation of action, but also for understanding the tool-action outcomes themselves. What remains unclear is what particular variables impact the perception of action and the understanding of action goals.

One variable that has been studied is the perspective of observed actions. It has been suggested that perspective encompasses not only visual objects in a scene, but also how concentration is focused in order to determine specific judgments about the environment (Lindgren, 2012). In this work, first and third person perspectives in a virtual world simulation were analyzed. Results indicated that there was a significant advantage in subject’s memory for tasks and task related elements when watching first person perspective simulations. Subjects also achieved higher accuracy during recall. How this applies to interpreting action based on other people’s movements that are typically in the third person perspective is still unclear. Mentally simulated actions from an egocentric perspective are considered visually and motorically familiar (Ni Choisdealbha et al., 2011; Conson et al., 2012) as this affords optimization of motor imagery and action encoding. Alternatively, the allocentric perspective may not be motorically familiar to oneself, and in order to process allocentric action, motor imagery may necessitate visual transformations. In Ni Choisdealbha et al. (2011), they showed that right and left-handed subjects were faster at judging hand stimuli in an egocentric orientation that corresponded to their own dominant hand. It was proposed that this effect was due to better utilization of visual and sensorimotor information to facilitate judgments in the dominant limb. In allocentric orientations, behavioral strategies shifted to “visual only” so that subjects could reorient the stimuli to align with “self” as a method for interpretation. This in turn suggests that subjects use a self-centered motor strategy to interpret action.

However, it is unclear how a subject’s handedness and the hand involved in seen actions may affect these results. In previous work, it has been shown that the left cerebral hemisphere is specialized for tool-use action (Raymer et al., 1999; Frey et al., 2005). Neuroimaging studies have shown left lateralization in right-handed participants for both left and right hand tool pantomime movements (Moll et al., 2000; Choi et al., 2001; Johnson-Frey et al., 2005; Bohlhalter et al., 2009; Cabinio et al., 2010). Further, left parietofrontal lateralization for performance of tool-use action was observed in left and right-handed subjects using their dominant hand (Vingerhoets et al., 2012). This evidence leads to the indication that damage to the left cerebral hemisphere resulting in ideomotor apraxia (which causes the inability to correctly perform tool-use and communicative gesture on command) should be a bilateral deficit (Wheaton and Hallett, 2007). Apraxia can be seen in both hands after left hemispheric damage, which suggests that the left hemisphere network controls skillful tool-use knowledge for both left and right hand movements (Heath et al., 2003).

**FIGURE 1:**
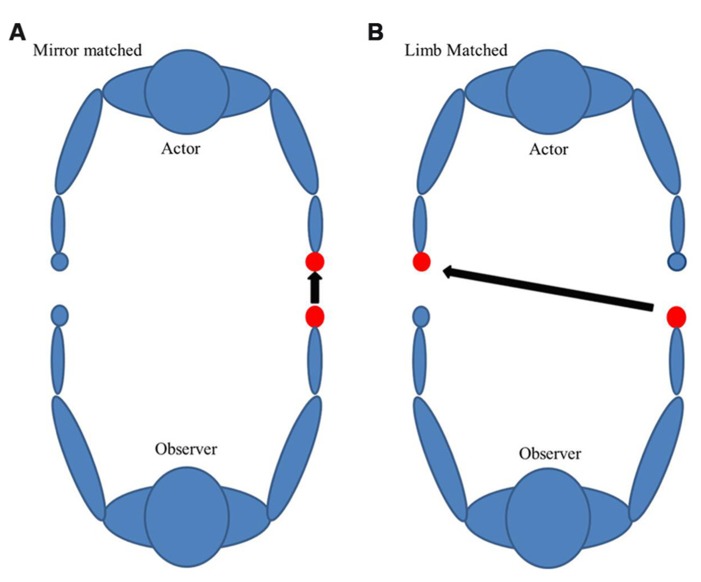
**There are two possible ways an action can be encoded from an allocentric perspective: (A) Mirror or **(B)** limb-matched.** The following figure is an example for a dominant right-handed subject.

It is worth considering that in left-handed subjects, there is a unique hemispheric dissociation which exists for motor planning of tool use (left parietofrontal) and primary motor cortices (right motor cortex). Whether this dissociation is disadvantageous to understanding action outcomes is a key goal in this work. It has been argued that encoding seen action utilizes a principle of motor resonance, where seen actions may be encoded in the observer’s motor system, perhaps using motor representations from the contralateral hemisphere of the seen arm (Gallivan et al., 2013). For actions seen in an egocentric (first person) perspective, limb-specific resonance is achievable. Under these circumstances, right-handed subjects watching a right-handed action would have no dissociation of motor planning and primary motor cortex. However, due to the diminished left lateralization of motor activation of left-handed action in right-handed subjects (Cabinio et al., 2010), there is the potential for some dissociation for right-handed subjects watching left-handed action. This assumes that action is encoded in the subject’s limb that matches the seen action. It is unclear what would happen in left-handed subjects, where seeing a right-handed action may bring tool-use activation and motor activation into the same hemisphere. Further, we frequently have to understand actions in daily living, and we commonly view them from an allocentric (third person) perspective. There are two possible ways an action can be encoded in the allocentric perspective in order to understand that action: limb-matched and mirrored-matched (**Figure 1**). Limb-matched is a biological-limb match to the subject. For example, for a dominant right-handed person it would be a right-handed allocentric action. Mirror-matched would occur when watching a matched dominant limb perform an action as if you were looking in a mirror (for a dominant right hand person it would be a left-handed allocentric action). According to prior studies, mirror-matched movements are less challenging to imitate because they are spatially compatible and do not require a shift of reference (Chiavarino et al., 2007). Other studies show that both right and left-handed subjects were faster in egocentric perspectives when looking at their dominant hands and faster in allocentric perspectives when looking at other’s non-dominant hands (Conson et al., 2010). Thus, mirror-matched may be advantageous in this paradigm.

The motivation of this study is to evaluate how perspective and handedness interact to understand and identify tool-action outcomes. Our hypothesis was that both left and right-handed subjects would identify action outcomes best from an egocentric perspective. When looking at stimuli from an allocentric perspective, identification of action outcomes would best occur in mirror-matched dominant limb for right and left-handed subjects. This study will help us better understand how we translate handedness and motor representations from different perspectives.

## MATERIALS AND METHODS

### SUBJECTS

Twenty right-handed subjects (7 males; average age, 22.8, SD, 3.0) and 19 left-handed subjects (11 males; average age: 21.6, SD, 2.2) participated in the study. All subjects were neurologically normal and had normal or corrected-to-normal vision. Handedness was evaluated by the Edinburg Handedness Inventory (Oldfield, 1971) with right-handed subjects having an average score of 82.54 (SD: 15.87) and left-handed subjects averaging -57.65 (SD: 26.81). If the handedness score was > + 40 then the subject was right-handed and if the score was <-40 then the subject was considered left-handed. If the subject was between +40 and -40 inclusive, the subject was considered ambidextrous and was excluded from the study. The maximum score is +/-100. The experimental procedure was approved by the Georgia Institute of Technology Institutional Review Board and consent was obtained from all participants prior to experiment.

### TRAINING

**FIGURE 2:**
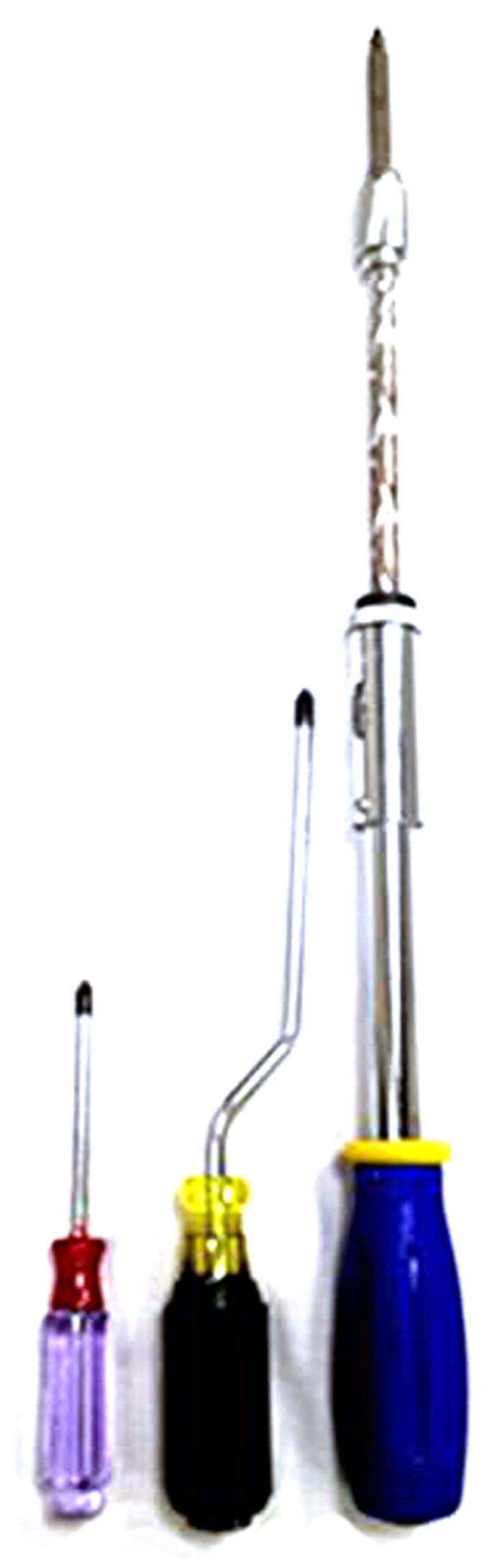
**A familiar twist screwdriver, a rotational (plumbers’) screwdriver, and a “Yankee” push screwdriver (from left to right)**.

Subjects were first trained on inserting and extracting tools on an upright stationary wooden board with screws protruding facing the subject. The subject had to use three different tools to perform the task, two were unfamiliar and one was familiar. Familiarity of the tools was confirmed verbally by subjects when prompted if they knew what each tool was. If they were familiar with an “unfamiliar” tool or unfamiliar with a “familiar” tool they were excluded from the study. The familiar tool was a twist screwdriver, while the unfamiliar tools were a push style “Yankee” screwdriver and a rotating (plumber’s) screwdriver being used by an actor (**Figure 2**). The use of multiple screwdrivers allowed us to maintain task and instruction consistency. These screwdrivers were particularly chosen because to use them, very different actions are required, but the action outcome is the same (insert or extract). The twist screwdriver uses a simple clockwise/counter-clockwise forearm rotation to insert or extract the screw. The push style screwdriver operates by pushing the driver handle that rotates the bit clockwise or counterclockwise based on the position of a toggle switch. The plumber’s screwdriver is similar to the twist, except that it demands circular rotation at the elbow to insert or extract the screw. The twist is the most familiar with push and rotational being the least familiar. Of these three, the push only has one action to insert or extract the screw (the other two require clockwise or counterclockwise rotation) and it is treated as a control image. A training board was placed in front of the subject’s visual field and was reachable at arm’s length. Participants used each of the three screwdrivers to insert five screws all the way into the board and then screw the same screws all the way back out to their initial starting position to obtain the motoric actions required to use each tool. Subjects were instructed to choose any five screws that were at a comfortable height for them to manipulate.

### STIMULI AND TASK

After all training was completed, subjects performed an action understanding task based on the trained tools. Subjects were seated comfortably in a chair and shown randomized action images of the three different tools on a 106.7 cm (42 inch) visual monitor (visual angle = 18.7°). Images were high-resolution grayscale images of either a right or left-handed instructor holding one of the previously mentioned tools in either an allocentric or egocentric perspective.

**FIGURE 3:**
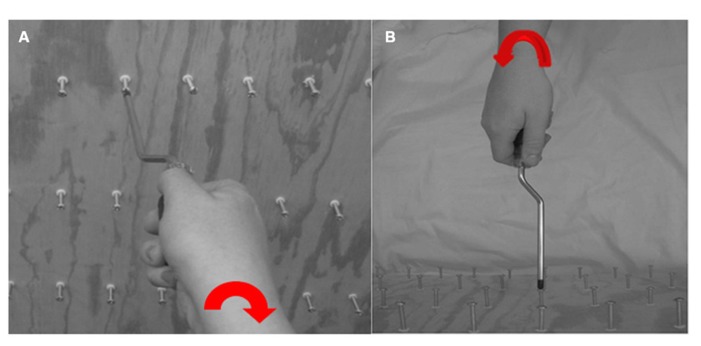
**The wooden board with screws that were mounted for subject training; (A) an exemplar image for right-handed egocentric rotating screwdriver driving a screw “in” and **(B)** an exemplar image for a left-handed allocentric rotating screwdriver pulling a screw “out”**.

**FIGURE 4:**
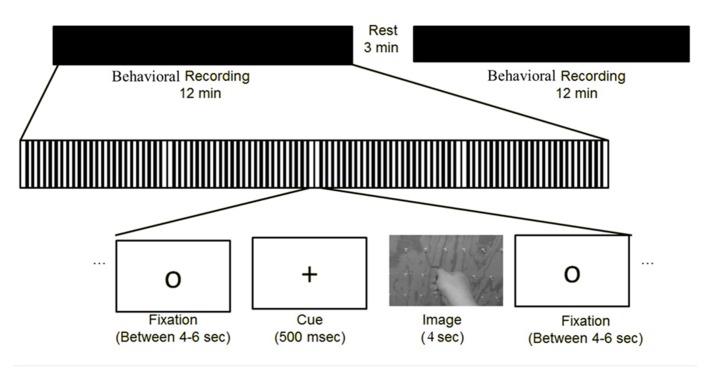
**Depicts the event-related experimental design of the study**.

While seated with a response pad comfortably in their hands, subjects were presented first with a circle (4–6 s), then a fixation cross which alerted subjects that the trial was about to start (500 ms), followed by the instructor-tool image (4 s). While the image (**Figure 3**) was on the screen, the subject was told the following: “The images on the screen will show you any of the tools you have just trained with, being used by either a left or right hand instructor, and can be shown either in an egocentric (as if you yourself are using the tool) or allocentric (as if you were watching me use the tool) perspective. On the image there will be a red arrow located on the wrist of the actor. Based on the direction of the arrow, you will need to simulate in your mind which way the hand is rotating, and answer if the hand is driving the screw into the board, or is it pulling the screw out of the board.” If they thought the actor was inserting the screw into the board, they were instructed to indicate by pushing the left button with their left hand on the response pad. If they thought the actor was extracting the screw, they were instructed to indicate by pushing the right button with their right hand on the response pad. Based on the stimuli presented, this afforded an equal number of responses with the left and right hands without bias to the response hand matching the stimulus hand (i.e., a correct response would equally occur for the same number of left or right hand image actions). The subject was instructed to answer as quickly and accurately as possible from the onset of the image. If the subject did not respond before the 4 s time period, the circle reappeared and no response was counted. There were 12 different image types. Each type was displayed twice in each of the two blocks that lasted approximately 13 min each (**Figure 4**). All images were presented in a pseudorandom order and correctness and latency of responses were recorded.

### ANALYSIS

Behavioral responses were recorded over two blocks of trials. All responses were recorded with Stim2 version 4.0 (Neuroscan 2003, El Paso, TX). Data sets were imported into Excel spreadsheets and organized by type into blocks. For each block, the response and latency average were calculated for each subject and every image type excluding any trials that the subject missed. Overall, there was no significant difference in missed trials for any image type (*p* = 0.685). All block averages were compiled into a grand average for each image type. Averages were then entered into IBM SPSS Statistics 19. A 4-way multivariate ANOVA (MANOVA) was computed with factors perspective (egocentric and allocentric) x hand of actor (left and right hand) x tool (traditional and rotational screwdrivers) x hand of subject (left and right-handed). Where appropriate, *t*-tests were used to identify interaction effects between the different image types. For *t*-tests, significance was assessed at *p* < 0.05 with Bonferroni correction for all comparisons.

## RESULTS

### LATENCY

**FIGURE 5:**
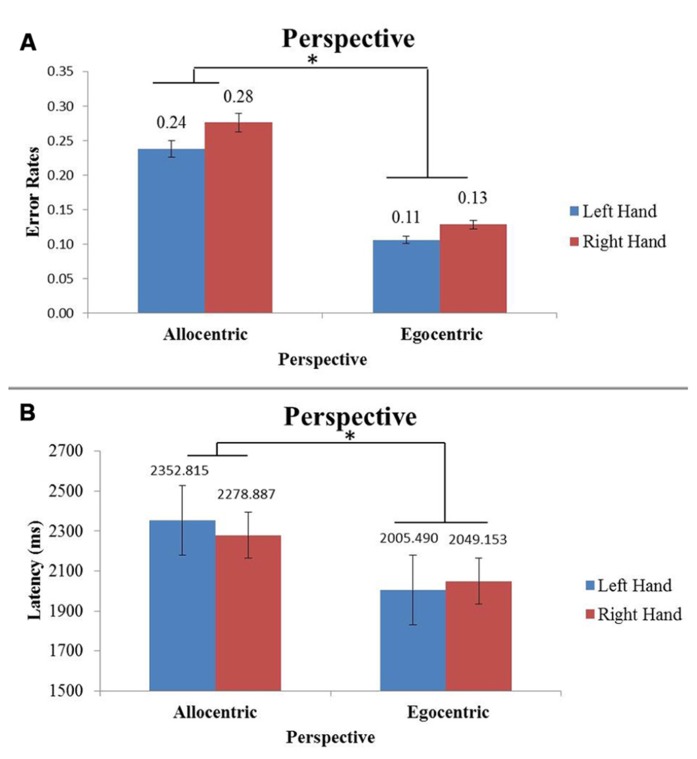
**Figure (A) shows average error rates for allocentric and egocentric images.** Figure **(B)** shows the average latency for allocentric and egocentric images. The x-axis represents perspective of the image separated by hand viewed. Perspective is statistically significant (*p < 0.05) between the error rate and latency graphs with egocentric images having the lowest latency and error rates.

For latency of response time, there was a main effect of perspective (*F*(1,304) = 33.66, *p* < 0.05) and of tool (*F*(1,304) = 9.23, *p* < 0.05). In **Figure 5A** it is shown that when subjects look at egocentric images, they respond significantly faster than if they were looking at an allocentric image. Looking at novel tool images, subjects respond slower when compared to familiar tools.

There were no other main or interaction effects regarding latency.

### ACCURACY

Accuracy (percent correct) was also evaluated for each image type. There was a significant main effect in percent correct due to perspective (*F*(1,304) = 37.44, *p* < 0.05), with the egocentric perspective having lower error rates (**Figure 5B**). There was a second main effect with respect to percent correct for hand of subject (*F*(1,304) = 8.31, *p* < 0.05), with right-handed subjects having lower error rates than left-handed subjects.

**FIGURE 6:**
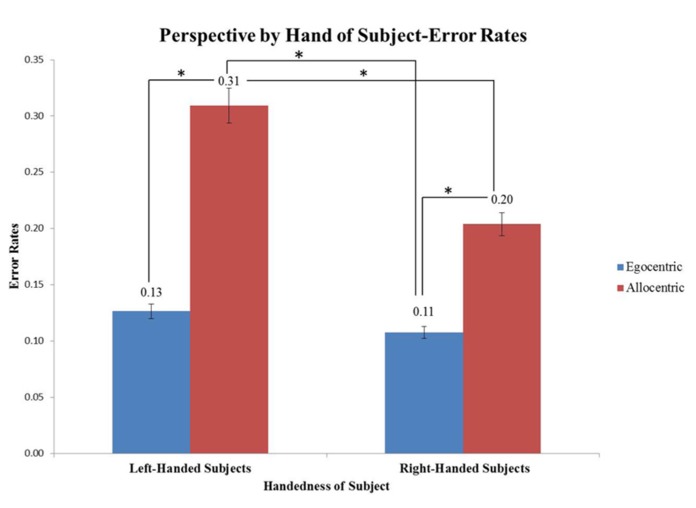
Graph depicts an interaction effect between perspective and hand of subject (*p < 0.05). Right-handed subjects looking at images in the egocentric perspective were more accurate at the task when compared to allocentric images (*p = 0.019). Overall, left-handed subjects looking at images in an allocentric perspective were significantly worse compared to all other conditions (*p < 0.05).

An interaction effect was seen for perspective x hand of subject (*F*(1,304) = 4.06, *p* < 0.05). Right-handed subjects looking at images in the egocentric perspective had significantly lower error rates compared to allocentric images (*p* = 0.019). Left-handed subjects looking at images in an allocentric perspective had the highest error rates overall compared to all the other conditions (**Figure 6**). An additional interaction effect was seen for tool x hand (*F*(1,304) = 4.88, *p* < 0.05), however when explored, there were no significant individual effects.

## DISCUSSION

Right and left-handed subjects were recruited in order to judge tool-use action outcomes while hand of instructor, perspective, and tool type used in the images were manipulated. Specifically, we sought to evaluate how perspective and handedness interact on a learned tool in order to accurately determine an action goal using a discrete motor task. In conformation of our first hypothesis, we found that egocentric perspective images had higher accuracy and faster latencies when compared to allocentric images. Our second hypothesis was refuted, as there was no effect of handedness of subject and limb performing the action. We will further discuss our findings based on the hypotheses presented.

### ALLOCENTRIC VERSUS EGOCENTRIC PERSPECTIVES

Our first hypothesis was that both left and right-handed subjects would be able to judge action best from an egocentric perspective. Results revealed there was a significant effect of accuracy and latency with respect to perspective for both right and left-handed subjects. The highest accuracy and fastest latency was found in the egocentric perspective for both sets of subjects, which supports our first hypothesis. These findings are in line with previous studies which suggest that action outcomes are best facilitated in an internal (egocentric) perspective, regardless of the hand being used (Conson et al., 2010; Lindgren, 2012; Oosterhof et al., 2012). Looking at previous neural studies, the left parietal lobe has been shown to be active in coding representations of the body, and the right parietal lobe is active for visuospatial orienting (Iacoboni et al., 1999; Watanabe et al., 2011). Specifically, Watanabe et al. (2011) studied right-handed subjects who viewed and then imitated limb-matched (“anatomical”) and mirror-matched (“specular”) images performing a finger touch task. The findings in this work suggested that the more dissimilar the actors hand was from the position of the participants, the more difficulty they had in interpreting the imitation task, and there was a corresponding notable increase in right posterior parietal cortex (PPC) activation. They suggested that the increase in activation was due to the demands of aligning visuospatial representations with kinesthetic signals from self and therefore it was more challenging to imitate the images. These findings could explain why our behavioral results showed effects of latency and accuracy, particularly disadvantageously in the allocentric perspective. Together, these authors suggest that when an action is observed in the allocentric perspective, it is possible that action resonates to either of the observer’s limbs as a technique to interpret action more readily. Although visual areas associated with mental rotation were not assessed, this could be a future direction to further explore the neural mechanisms driving the behavioral effect.

### EFFECTS OF HANDEDNESS IN ALLOCENTRIC PERSPECTIVE

Our second hypothesis was that in allocentric perspectives, optimal action prediction would align best in mirror-matched dominant limb for right-handed and left-handed subjects. Handedness of subject showed an effect on accuracy, where right-handed subjects were significantly more accurate in the task than left-handed subjects overall. However, neither right nor left-handed subjects showed behavioral effects to the allocentric actions performed with a mirrored or matched hand, which does not support the second portion of our hypothesis. We studied action prediction by testing if the ability for resonance to occur may be impacted in a limb specific way. In action perception, according to the ideomotor theory, a subject’s motor system and the associated action representations are activated when perceiving action from another person (Massen and Prinz, 2009). Perceiving body movements and corresponding remote goals influences how those actions are understood. Functional affordances include all possible tool-based goal directed actions that best “afford” a desired action goal (Mizelle et al., 2013). In this work, we proposed that functional affordances are proposed to be critical for the ability to simulate action and understand all possible action outcomes. Importantly both body movements and action goals have a bidirectional association in order for the perception of action to trigger action in the observer (Massen and Prinz, 2009; Paulus, 2012). If the perception of action in an observer comes from bidirectional understanding of movements and goals, then mapping all seen action to the dominant or non-dominant limb in an allocentric perspective could facilitate action understanding. Although allocentric actions showed no bias to either limb for our behavioral study, Conson et al. (2010) did in fact see a limb bias in the allocentric perspective. This could be due to different experimental demands between the paradigms where our study was focused on action outcome and Conson et al. (2010) was focused on hand laterality and mental rotation. Future neurophysiological studies will further evaluate specific neural mechanisms that may relate to activation of left or right sensorimotor areas in a similar task.

When compared to right-handed subjects, left-handed subjects were significantly less accurate when judging the outcomes of allocentric images. The decreased accuracy for left-handed subjects on the allocentric images could be due to an asymmetrical lateralization of encoding action and motoric dominance in the brain, which may interfere with translating allocentric limb action outcomes within their own motor system. In prior work (Frey et al., 2005), left and right-handed callosotomy patients were studied in order to understand hemispheric specialization for tool-use. The left-handed patient performed worse at demonstrating tool-use actions with the dominant left hand compared to their right hand, but the right-handed patient performed best with the dominant right hand and worse with the left. These results indicate that the left hemisphere is specialized for tool-use information. This idea has been well validated in human neuroimaging experiments (Vingerhoets et al., 2012). For left-handed people (because the right hemisphere controls their dominant hand) a challenge is presented when trying to access tool representations from the opposite (left) hemisphere. However, performance of tool-use actions was not a disadvantage in their right-handed callosotomy patient. If tool-use information is stored in the left hemisphere for both right and left-handed people, then it is possible that because right-handed people have a dominant left motor hemisphere (creating a hemisphere match), they would have an advantage when interpreting action outcomes in our study. Extending these concepts, these results could suggest the reason left-handed subjects perform significantly worse in allocentric action outcome interpretation is because when they view the images they utilize an additional mechanism that is needed to facilitate coordination of information across the hemispheres. Specifically, we propose that when action is seen in the allocentric perspective, left-handed subjects have an additional demand of utilizing left hemisphere action encoding along with right hemispheric motor and visuospatial rotations to comprehend action outcomes (Watanabe et al., 2011). Importantly, right hemispheric visuospatial rotation may relate to right-handed subjects performing worse on allocentric versus egocentric actions (**Figure 6**). Why this affects accuracy, but not latency is worth consideration in behavioral and neurophysiological studies to understand aspects of decision delay versus decision accuracy in similar tasks.

### EFFECTS OF LATENCY VERSUS ACCURACY

The finding that latency was significantly increased for allocentric images contributes to previous research that states allocentric images are harder to interpret compared to egocentric images (Ni Choisdealbha et al., 2011; Zhou et al., 2012). However, latency effects did not persist through any other variable in this study. Given the difficulty of the task, there could possibly be no other latency differences because all images are moderately difficult, which would extend reaction time and ultimately interfere with accuracy due to the time constraints on response time. We removed the missed trials for each condition, which was 27.5% of trials in each condition (there was no significant difference in missed trials for any image type (*p* = 0.685), which suggests the task was equally difficult for all stimuli. Previous studies in our lab involving affordance have shown effects of action encoding in the latency domain but not in the accuracy domain (Borghi et al., 2012). Whether increasing the time constraint on response interval or reducing the difficulty of action images would alter latency effects is an issue to be investigated in future research.

### ALTERNATIVE EXPLANATIONS

There is other existing evidence that would suggest it is possible that right and left-handed subjects have different strategies when it comes to interpreting action. Ni Choisdealbha et al. (2011) suggested that right-handed subjects rely primarily on sensorimotor mental rotation. On the other hand, left-handed subjects could depend initially on visual analysis and/or pictorial strategies followed by a mental rotation strategy.

Work has also been done to evaluate patients with frontal lesions on similar tasks (Chiavarino et al., 2007). The patients were asked to imitate mirror-matched or limb-matched stimulus. They discovered that patients had a selective deficit for imitating limb-matched responses which suggests that executive function of the frontal lobes drives the system to visually rotate the frame of reference in order for them to imitate the stimulus. They suggest that the imitation capacity was damaged for these particular patients. If this theory is true, then in our healthy population, left and right-handed subjects would have had a similar deficit when judging allocentric images. Although this is a valid explanation, we believe it is unlikely due to higher order executive function, but rather differences in the motor system. A limitation of their study was that they did not separate the patients into left and right sided brain lesion groups and they also had diverse locations where the lesions were located within the frontal lobe. Apraxia in left-handed patients with left or right hemisphere damage has been evaluated in a recent study by Goldenberg (2013). He found that in left-handed patients, apraxia can occur as a result of damage to either the left or right hemisphere. Apraxia after left hemispheric damage (dissociating from manual dominance) may be explained as result of damage to the praxis relevant networks which remain in the left hemisphere. However, apraxia after right hemispheric damage could be explained as result of damage to a unique co-localization of praxis skills and spatial processing within the right hemisphere. Such findings could argue for a stronger bilateral organization of praxis control in left handed compared to right handed subjects.

### LIMITATIONS

A limitation of the current study is that it is difficult to recruit left-handed subjects that are extremely left hand dominant. Most tools are designed for right-handed people, thus left-handers acclimate and become slightly more ambidextrous for some skilled unimanual tasks. This effect could confound the interpretation of potential hemispheric dissociations, as strength of left-handedness has been shown to augment the strength of right hemispheric laterality (Cabinio et al., 2010). Ambidextrous subjects were excluded from the present study, but left-handed subjects had a lower overall hand dominance score when compared to the right-handed subjects on the Edinburg Handedness Inventory scale. Each individual subjects score was, according to the Edinburg Handedness Scale, beyond the ambidextrous range.

Another limitation is although we were not seeking to understand the learning of new tools, a new tool was incorporated into the study in order to obtain selection of tools that had the same action of “screwing.” Our study utilized direct training for all tools presented and there was no effect of accuracy for novel versus familiar tool observed. There was an effect on latency, with novel tools overall having an increased latency compared to that of familiar tools. We did not expect to see a difference behaviorally between tool types due to previous work indicating neural networks were the same; however, the addition of a neural study would be able to confirm this.

## CONCLUSION

The current findings provide insight into how action-goals are encoded and interpreted by left and right-handed subjects. We have demonstrated that encoding of action of left and right-handed actors is not necessarily differentially encoded in left or right-handed subjects in a way that would demonstrate behavioral differences. We have shown there is a benefit in representation of actions encoded in the egocentric perspective. While the ideomotor theory can explain much of why this occurs, it is still unclear as to why left-handed subjects viewing allocentric action showed the pronounced deficit from other combinations of handedness and perspective. Future research may determine the specific neural mechanisms that drive these results by collecting neurophysiological data focusing on motor lateralization effects, which is currently underway.

## Conflict of Interest Statement

The authors declare that the research was conducted in the absence of any commercial or financial relationships that could be construed as a potential conflict of interest.
